# Cardiorespiratory and metabolic stress responses to acute high-intensity interval training anchored to critical power or maximal heart rate

**DOI:** 10.1038/s41598-025-28231-y

**Published:** 2025-12-29

**Authors:** Jack Bone, Douglas L. Richards, Martin J. Gibala

**Affiliations:** 1 Department of Kinesiology, McMaster University, Hamilton, ON Canada; 2 Department of Medicine, McMaster University, Hamilton, ON Canada

**Keywords:** Exercise prescription, Metabolic thresholds, Metabolic variability, Exercise intensity domains, Cardiology, Health care, Medical research, Physiology

## Abstract

High intensity interval training (HIIT) involves repeated bouts of relatively hard work, commonly at intensities eliciting ≥ 80% of maximal heart rate (HR_max_), interspersed with recovery periods. Anchoring intensity to HR_max_ can elicit a wide range of acute metabolic responses to exercise. Expressing intensity relative to metabolic thresholds such as critical power (CP) may reduce this variability. We therefore examined whether anchoring HIIT to CP reduced variability in change in [blood lactate] (ΔBLa^−^) compared to HR_max_-based approach. Nineteen adults aged 23 ± 4 years completed two 4 × 4-min HIIT trials in a randomized, crossover manner at intensities equal to CP + 10% of work prime (CP_HIIT_) or ≥ 80% HR_max_ (HR_HIIT_). Variability in [ΔBLa^−^] from rest to exercise was not different between CP_HIIT_ and HR_HIIT_ (1.37 (0.42–1.62) vs. 1.32 (0.77–1.97) mM; *p* = 0.75). Workload was higher in CP_HIIT_ vs. HR_HIIT_ (192 ± 39 W vs. 180 ± 43 W; *p* = 0.001), as was exercise oxygen consumption, ventilation, respiratory exchange ratio, heart rate, and rating of perceived exertion (all *p* < 0.05). A CP-based HIIT protocol did not reduce variability of change in [ΔBLa^−^] compared to a traditional approach anchored to %HR_max_. However, anchoring HIIT intensity to CP resulted in participants achieving higher workloads, eliciting higher cardiorespiratory and perceived stress which could translate to divergent training-induced responses.

**Supplementary Information:**

The online version contains supplementary material available at 10.1038/s41598-025-28231-y.

## Introduction

High intensity-interval training (HIIT) is characterized by repeated bouts of relatively hard work interspersed with recovery periods of rest or light exercise^1^. Like all forms of exercise, HIIT can vary widely depending on the essential elements of the FITT principle: frequency, intensity, time, and type^2,3^. Manipulating frequency, time, and type is relatively straightforward and mainly involves changing the number of exercise sessions per week, the amount of time spent exercising per session, or the mode of exercise^4^. The exercise intensity variable, however, is more nuanced as it can be characterized using a wide array of methods^5^.

The classification of exercise intensity can be broadly contextualized within a health or performance context^5^. Intensity prescription based on “traditional” physiological anchors such as maximal heart rate (HR) is common in a health and general fitness context, with HIIT often defined as eliciting ≥ 80–100% of HR_max_^3,6^. In contrast, intensity prescription in a performance context is typically based on a three-domain model with the descriptors moderate, heavy, and severe^4,5,7^. These domains are in turn anchored to “metabolic” thresholds including those based on work rate (i.e. power or speed) or blood lactate (BLa^−^). HIIT is typically characterized as an intensity in the severe domain^5^, which is can be demarcated from the heavy domain by critical power (CP). There is a finite amount of work that can be completed above CP in the severe intensity domain, as represented by the power-duration curvature constant of the CP concept, called work prime (W′)^7^. Exercise above CP is associated with the attainment or near attainment of maximal oxygen uptake ($\dot{V}$O_2max_), the accumulation of metabolic by-products and acidosis, and eventual exhaustion^8–11^. Robust evidence supports the validity as this boundary between the heavy and severe exercise domains in terms of eliciting distinct physiological responses^8–10^ that distinguish relatively sustainable from non-sustainable exercise.

There is considerable interindividual variability in the percentages of traditional physiological anchors such as HR_max_ at which metabolic thresholds occur^12–14^. Indeed, fixed percentages of HR_max_ can exhibit “domain-overlap”. This means that two individuals can exhibit diverse physiological responses when exercising at a given %HR_max_, indicative of work in distinct metabolic domains. One range of domain overlap is ~ 80–90% HR_max_, with individuals exhibiting physiological responses characteristics of both the heavy and severe domains^12–14^. This range notably corresponds to the lower boundary of HIIT as commonly defined^12,14^. Performing HIIT using a HR_max_-based anchor may thus exacerbate the variability in physiological responses to this form of exercise. In contrast, anchoring HIIT intensity to a metabolic threshold such as CP may reduce such variability by ensuring that individuals are exercising within a given exercise domain and thus experiencing a similar metabolic stress^4^. Some evidence supports the notion of more homogeneous responses when exercise is prescribed relative to metabolic thresholds such as the first and second ventilatory thresholds ($\dot{V}$T1 & $\dot{V}$T2) as opposed to maximal physiological anchors^15–17^. This includes some work specifically focused on HIIT by Meyler and colleagues^17^, who reported reduced variability in [BLa^−^] after a HIIT protocol anchored to CP compared to maximal oxygen uptake $\dot{V}$O_2max_ in healthy, active adults.

The primary purpose of the present study was to compare the acute physiological responses to a common HIIT protocol^18,19^, in which exercise intensity is prescribed relative to a traditional anchor (i.e. % HR_max_), and a metabolic threshold based on CP. Building on the work of Meyler et al.^17^, the primary outcome was the variability in the acute exercise-induced change (Δ) in [BLa^−^]. We hypothesized that the variability of the [ΔBLa^−^] would be lower in the CP- compared to %HRmax-based protocol. Secondary outcomes included measures of HR, $\dot{V}$O_2_, ventilation ($\dot{V}$_E_), and perceived exertion (RPE) to characterize the cardiorespiratory and perceived stress of the two trials.

## Materials and methods

### Participants and ethical approval

**Fig. 1:**
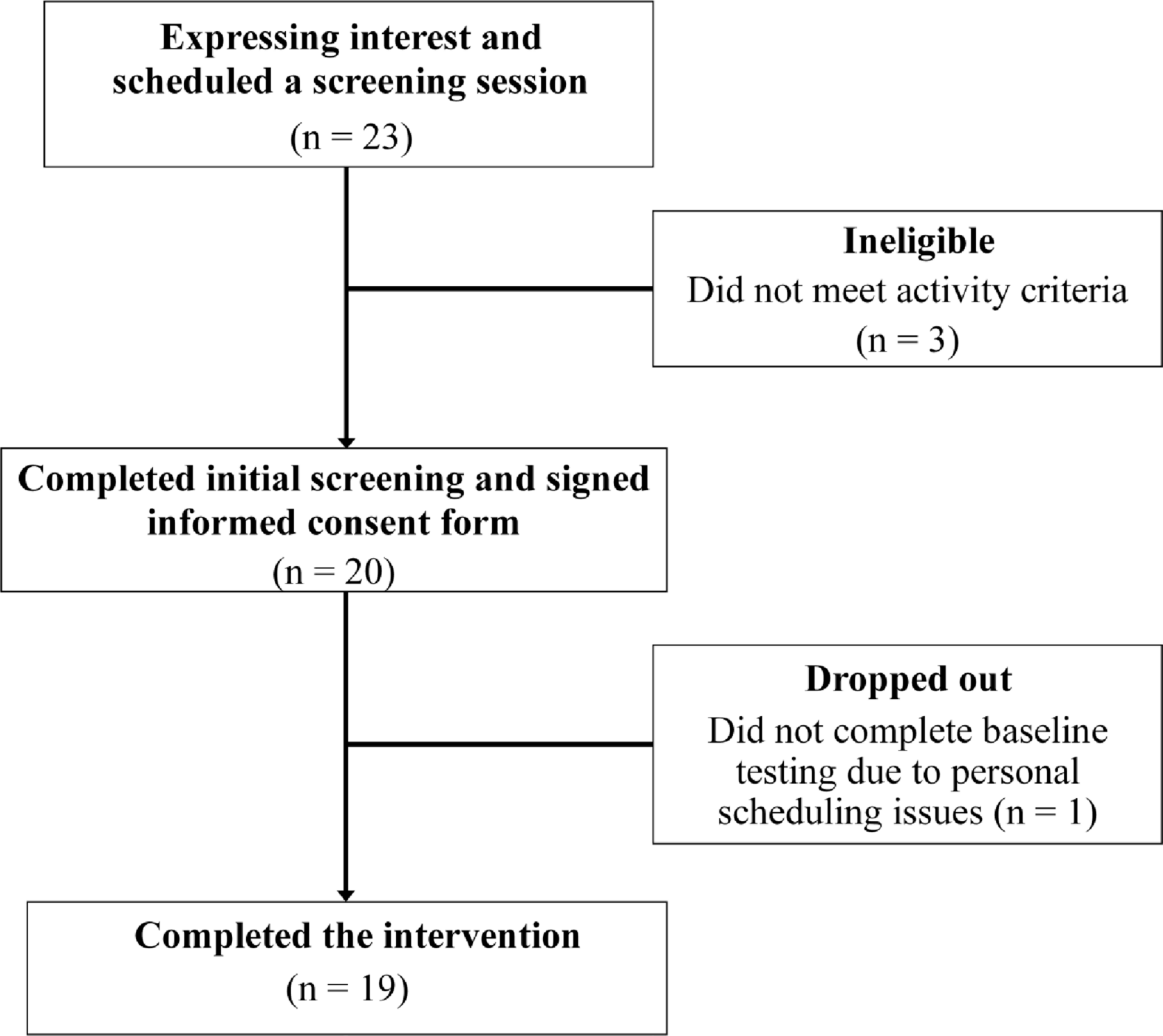
Study CONSORT diagram.

The inclusion criteria were being apparently healthy, active adults aged 18–35 who fell within either Tiers 1 or 2 of the participant classification framework outlined in McKay and colleagues^20^. Tier 1 encompasses those who achieve ≥150 min of moderate to vigorous aerobic physical activity per week, which was assessed based on self-report using the Canadian Society for Exercise Physiology Get Active Questionnaire (https://csep.ca/2021/01/20/pre-screening-for-physical-activity/). Tier 2 includes those training ~ 3 times per week with a purpose to compete. All participants were engaged in cycling as a part of their habitual exercise regimen. Sample size was estimated via a calculation performed in G*Power (version 3.1.9.7) for a repeated measures analysis of variance (ANOVA) with two factors and two levels. It determined that a total sample size of 15 provided 80% power to detect a difference at an alpha level of 0.05 with a large effect size *f* of 0.40. To preserve power, 20 participants were initially recruited. One participant was lost to follow up, leaving *n* = 19 who completed all trials (9 males, 10 females; Fig. 1). Participant characteristics are summarized in Table 1. Participants were recruited from McMaster University and the surrounding community with information about the study being shared using printed posters, word of mouth, and social media posts. Interested individuals were informed of the study requirements and provided with an electronic copy of the informed consent form. Individuals who satisfied the inclusion criteria and wished to participate provided written informed consent and were subsequently recruited into the study. The study protocol was approved by the Hamilton Integrated Research Ethics Board and all methods were performed in accordance with the relevant guidelines and regulations. The study protocol was also registered on Open Science Framework prior to data collection (osf.io/s68mh).

**Table 1:** Participant characteristics.

	Males (*n* = 9)	Females (*n* = 10)	Group Combined (*n* = 19)
Age (years)	25 ± 5	21 ± 3	23 ± 4
Mass (kg)	80.5 ± 9.0	61.6 ± 6.0	70.6 ± 12.2
$\dot{V}$O_2peak_ (L·min^−1^)	4.03 ± 0.71	2.63 ± 0.35	3.29 ± 0.90
$\dot{V}$O_2peak_ (ml·kg^−1^·min^−1^)	50.1 ± 7.5	42.7 ± 4.5	46.2 ± 7.0
HR_max_ (bpm)	185 ± 10	183 ± 8	184 ± 9
PPO (W)	343 ± 40	255 ± 30	296 ± 57
Critical power (W)	212 ± 29	161 ± 22	185 ± 34
Critical power SE (W)	5 ± 3	3 ± 2	4 ± 3
W′ (W)	21,605 ± 5893	11,192 ± 3741	16,215 ± 7139
W′ SE (W)	2338 ± 1422	1441 ± 1065	1866 ± 1295

All data are mean ± SD (*n* = 19). $\dot{V}$O_2pea_ = peak oxygen uptake; HR = heart rate; PPO = peak power output at the end of the $\dot{V}$O_2peak_ test; HR_max_ = maximal exercise heart rate; SE = standard error; W′ = work prime.

### Overview of experimental design

Participants made nine to eleven visits to the laboratory over a period of four to five weeks depending on the number of tests required to determine CP. Participants initially made two visits to determine $\dot{V}$O_2peak_ as well as peak power output (PPO), HR_max_, and $\dot{V}$T1. This was followed by a series of maximal, constant load tests over several days to determine CP^9,10,21^. Participants then made two visits to familiarize themselves with the workloads and relative intensities to be used for the experimental trials. The two experimental trials were conducted in a randomized, and counterbalanced manner, and were performed at the same time of day (± 1 h) for a given participant and separated by a minimum of 48 h. The protocols differed in that one was performed at an intensity equal to CP + 10% W′ and the other at the intensity that elicited a peak exercise HR (HR_peak_) of 80–90% HR_max_ as verified during familiarization testing. The workload of the CP trial was anchored relative to W′ depletion to further individualize exercise intensity prescription. While previous research has prescribed CP-based exercise intensity as a percentage of individual CP (i.e. 110% CP)^17^, this method does not attempt to standardize the depletion rate of individual W′. Participants were asked to maintain their habitual physical activity and diet throughout the course of the experiment.

### $\dot{V}$O_2peak_ tests

Body mass and height were measured prior to the completion of a ramp exercise test on a cycle ergometer (Lode Excalibur Sport V 2.0, Groningen, The Netherlands). Following a 3-minute warm-up at 50 W, a ramp protocol was applied with a linear workload increase of 1 W every 2 s. Participants were instructed to choose a cadence between 70 and 90 rpm and maintain it for as long as possible until volitional exhaustion or cadence fell below 30 rpm. A recovery phase was performed at 50 W. Gas exchange and respiratory variables were continuously measured using a metabolic cart (Quark CPET metabolic cart, COSMED, Italy), and HR was also measured continuously (Polar A3, Finland). Data was averaged over 10-s intervals and $\dot{V}$O_2peak_ was defined as the highest 30-s average over three consecutive intervals. HR_max_ was determined as the highest HR achieved during the test. Data-based cut-offs for age-stratified secondary exhaustion criteria based on peak respiratory exchange ratio (RER; ≥1.13) and age-predicted maximal HR (208-(0.7*age) ≥ 93%), along with the attainment of volitional exhaustion, were used to assess whether the test involved maximal effort^22^, which was considered achieved if at least two of the three criteria were met. The peak RER cut-off and age-predicted maximal HR cut-off were achieved in 76% (29/38) and 74% (28/38) of tests, respectively, while volitional exhaustion was attained in 100% of tests (38/38). The term $\dot{V}$O_2peak_ is used throughout as the attainment of a “true” maximal oxygen uptake ($\dot{V}$O_2max_) was not demonstrated in all participants, nor was a verification phase employed. Baseline $\dot{V}$O_2peak_ is reported as the mean of both baseline $\dot{V}$O_2peak_ tests. $\dot{V}$T1 was visually evaluated from $\dot{V}$O_2peak_ respiratory data via the ventilatory equivalent ($\dot{V}$E/$\dot{V}$CO_2_ and $\dot{V}$E/$\dot{V}$O_2_), the partial pressure of O_2_, and the V-slope methods. These values were combined and averaged for determination of $\dot{V}$T1^11,23^.

### Critical power tests

Constant load tests were conducted on a cycle ergometer (Lode) and began with a 5-minute warmup at 50 W. Workload was immediately increased to the target power output for the test. Participants were blinded to the workload and elapsed time of the test. Participants were instructed to use the same cadence as for the $\dot{V}$O_2peak_ tests. Task failure during each test was defined as cadence falling > 10 rpm below the chosen cadence for > 5 s. Cadence was displayed throughout the trials. Strong verbal encouragement was provided throughout each test. All tests were performed at workloads between those at the determined $\dot{V}$T1 and PPO in the baseline $\dot{V}$O_2peak_ tests. Specifically, the first assigned workload was randomly set to between Δ90% (with Δ representing the difference in wattage between $\dot{V}$T1 and PPO) and Δ50%, and workloads for all subsequent tests were adjusted depending on the outcome of the prior test. CP was then calculated using the following three equations: the linear work-time model (Eq. 1), the inverse linear model (Eq. 2), and the hyperbolic model (Eq. 3)^9,10,21^.

1 $$W=CP\cdot t+W^{\prime}$$

2 $$P=W^{\prime} \cdot (1/t)+CP$$

3 $$t=W^{\prime}/(P-CP)$$

Within these equations, P represents workload, W represents the total work completed, and t represents the total duration of the trial in seconds. The CP estimate from the equation that yielded the highest coefficient of determination (R^2^) and lowest standard error (SE) was selected^21^. If all equations yielded a SE >5% after three bouts, up to two additional trials were performed at intensities between $\dot{V}$T1 and $\dot{V}$O_2peak_ until at least one equation provided an SE ≤ 5%^21^. All workloads were chosen to ensure a range of times to exhaustion between 2- and 15 min. This method of estimating CP is consistent with the gold standard approach^9,10,21,24,25^. CP was determined with 3 (*n* = 16), 4 (*n* = 2), and 5 (*n* = 1) trials using the linear work-time model (*n* = 19; R^2^ average 0.999 ± 0.001). Mean times to completion for the longest and shortest bouts were 691 ± 119 s and 217 ± 47 s, respectively. Once CP and W′ were determined, the intensity required to deplete 10% W′ within 4 min was determined by rearranging the determination model into the following format (Eq. 4), where t is equal to 240 s:

4 $$P=CP+(W^{\prime}*0.10)/t$$

### Familiarization trials

**Fig. 2:**
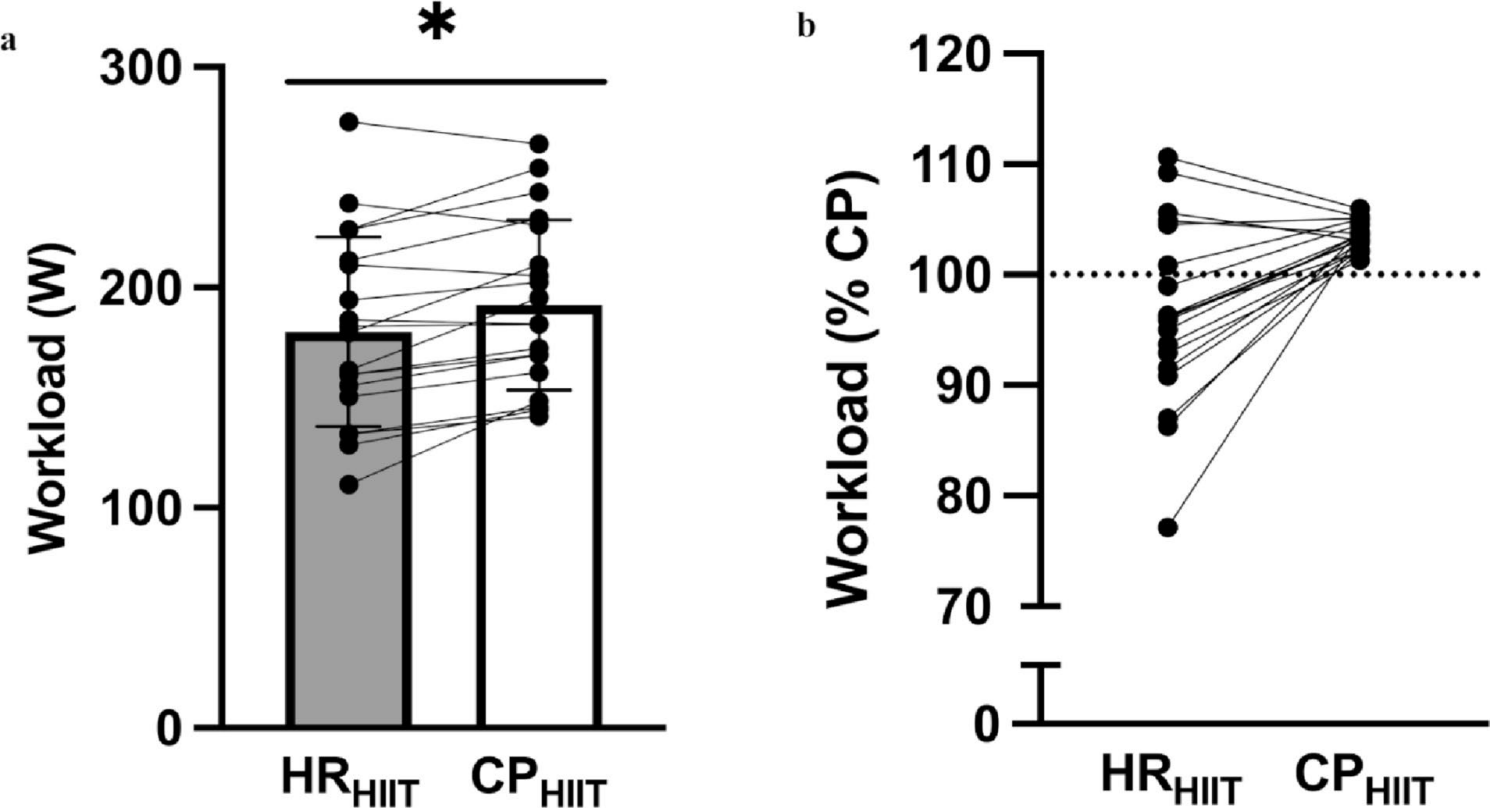
Workload data. (**a**) Mean workload during each trial. (**b**) Distribution of participants’ prescribed HIIT workloads relative to individual CP between conditions. Data in (**a**) are mean ± SD (*n* = 19). HR_HIIT_ = heart rate-based experimental condition; CP_HIIT_ = critical power-based experimental condition. ^*^p < 0.05.

Participants completed two single-bout HIIT sessions, one for each protocol. The purpose of these sessions was to familiarize participants with the protocols and to determine if the selected workload for the HR based protocol was appropriate. In both sessions, participants completed a 5-minute warm-up at 50 W, a single 4-min HIIT bout, and a 5-minute cool down at 50 W. For the HR-based condition (HR_HIIT_), the workload was set to 60% of PPO and intended to elicit a peak exercise HR > 80% and < 90% of HRmax by the end of the 4-min bout. If the HR elicited was ≤ 80% or ≥ 90% of maximum, the workload was adjusted prior to the experimental trials. An adjustment was needed for 14 participants, with 7 requiring an increase in workload (mean change of 9 ± 7 W) and 7 requiring a decrease (mean change of – 11 ± 13 W) to achieve the desired %HRmax range. The mean workload adjustment ranged from − 38 to 23 W compared to the initial workload setting that corresponded to 60% PPO. The workload for the CP condition (CP_HIIT_) was set at an intensity above CP meant to exhaust 10% of W′ when maintained for 4 min (CP + 10% W′). Mean workload in CP_HIIT_ and HR_HIIT_ were 192 ± 39 W and 180 ± 43 W, respectively (Fig. 2a). Workload in CP_HIIT_ was higher than that of HR_HIIT_ (*p* = 0.0013; Fig. 2a). Relative to individual CP, the mean workload in HR_HIIT_ was 97% CP (77–111%), while that of CP_HIIT_ was 103% CP (101–106%). A visual representation of workload distribution relative to CP is depicted in Fig. 2b.

### Experimental trials

On the day before each experimental trial, participants were asked to refrain from strenuous exercise and avoid consuming alcohol. Upon arrival at the laboratory, they rested quietly for 5–10 min before a capillary blood sample was obtained and measured in duplicate using a point of care analyzer (Nova Biomedical Xrcise Lactate Plus, Waltham, USA). After a 5-minute warm-up at 50 W, participants performed four, 4-minute bouts of exercise interspersed with 3 min of recovery at 25 W. Participants were asked to maintain their preferred cadence from the CP determination trials and keep it consistent throughout each 4-minute bout. HR was monitored continuously during exercise (Polar A3, Finland). Mean and peak HR was determined for the entire period of exercise and each 4-minute bout. A metabolic cart with an on-line gas collection system (Quark CPET metabolic cart, COSMED, Italy) was used to determine $\dot{V}$O_2_, $\dot{V}$CO_2_, $\dot{V}$_E_, and RER over the first 4-minute bout and last 4-minute bout. RPE was assessed with the 20-point Borg scale at the end of each bout^26^. Immediately upon the completion of the final bout, a second capillary blood sample was obtained. Participants then cooled down at 50 W.

### Statistical analyses

Normality was assessed with a Shapiro Wilk’s test, and if not normal, tested for lognormality. If data were lognormal, variables were log transformed before testing, and if not, non-parametric tests were performed. Analyses revealed that while some data were non-normal, all were associated with variability analyses. However, as these variables were already to be assessed using a Levene’s test (a non-parametric test itself), which is robust to deviations from normality, no data transformation or non-parametric alternative was required. A Levene test was used to measure variability in the primary outcome, [ΔBLa^−^] from rest to exercise, as well as all other measures. This is done by determining the absolute deviations of each data point from the condition mean and then comparing these deviations across conditions. [BLa^−^] and all other exercise data including HR, $\dot{V}$O_2_ across both the entire 4-minute bouts as well as in the final 30 s, $\dot{V}$_E_, and RER were compared using a two-way (condition by time) repeated measures ANOVA. A two-tailed paired t-test was used to analyze power output data as well as net [ΔBLa^−^] between CP_HIIT_ and HR_HIIT_. Significant main effects and interactions were further analyzed with a Tukey’s post hoc test and Fisher’s LSD. Participants missing a single data point (i.e. from one condition only) were included in the analysis, but the data were analyzed with a mixed effects model. This applied to the mean and peak HR data sets, in which a single data point was missing for one participant in Bout 2 of CP_HIIT_. Statistics were performed with SPSS Statistics (v. 29, IBM, Armonk, NY, USA) and Prism 10 (Graphpad, San Diego, CA, USA) with significance accepted as *p* < 0.05. Normal data are presented as mean ± standard deviation, and non-normal data as median (interquartile range). Effect sizes are presented as Cohen’s *d*_*z*_ and partial eta squared (η_p_^2^) for repeated-measures ANOVA unless stated otherwise.

## Results

### Blood lactate data

**Fig. 3:**
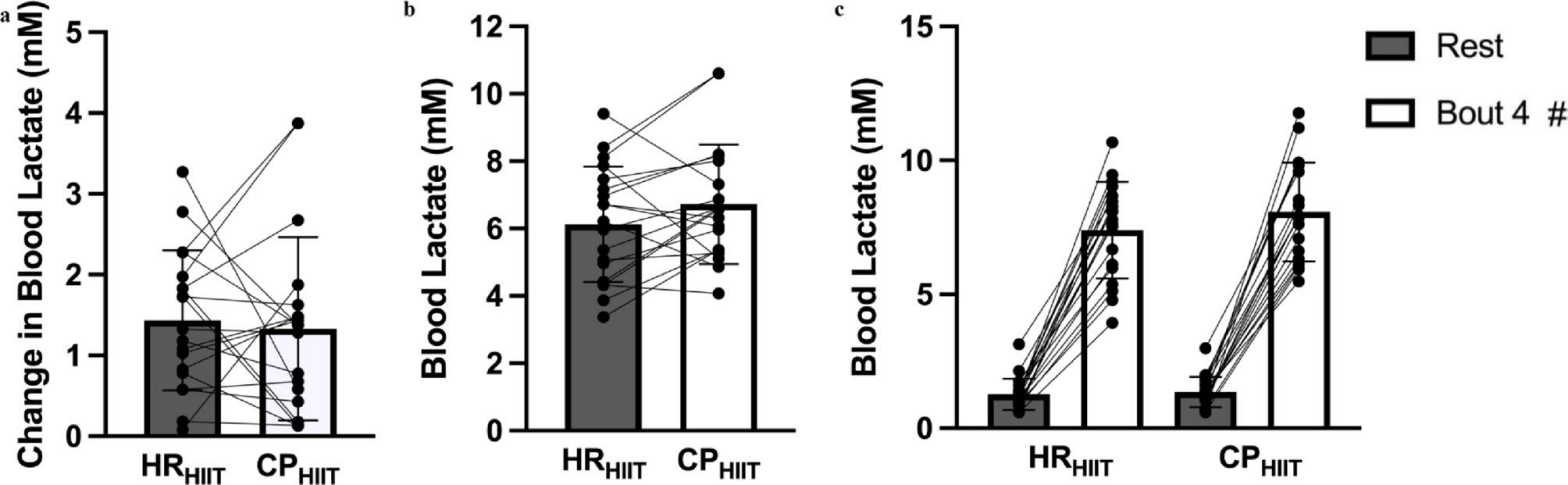
Capillary blood lactate data. (**a**) Variability of change in [blood lactate], determined from the net change in [blood lactate] from rest to the end of exercise (i.e. after Bout 4). (**b**) Absolute blood [lactate] accumulation between rest and the end of exercise. (**c**) Blood [lactate] at rest and at the end of exercise. Data for (**a**) is presented as median (interquartile range), and (**b**) and (**c**) are mean ± SD (*n* = 19). HR_HIIT_ = heart rate-based experimental condition; CP_HIIT_ = critical power-based experimental condition. ^#^*p* < 0.05 vs. Rest, main effect for time.

The variability of [ΔBLa^−^] was not different between CP_HIIT_ and HR_HIIT_ (1.37 (0.42–1.62) vs. 1.32 (0.77–1.97) mM; *p* = 0.75, *d*_*z*_=0.09, Fig. 3a). Net [ΔBLa^−^] was also not different between conditions (6.72 ± 1.78 vs. 6.13 ± 1.71 mM for CP_HIIT_ and HR_HIIT_, respectively; *p* = 0.10, *d*_*z*_=0.39, Fig. 3b). While [BLa^−^] increased from rest to exercise (main effect of time, *p* < 0.0001, η_p_^2^ = 0.99), the change was not different between conditions (*p* = 0.09, η_p_^2^ = 0.22; Fig. 3c).

### Cardiorespiratory data

**Fig. 4:**
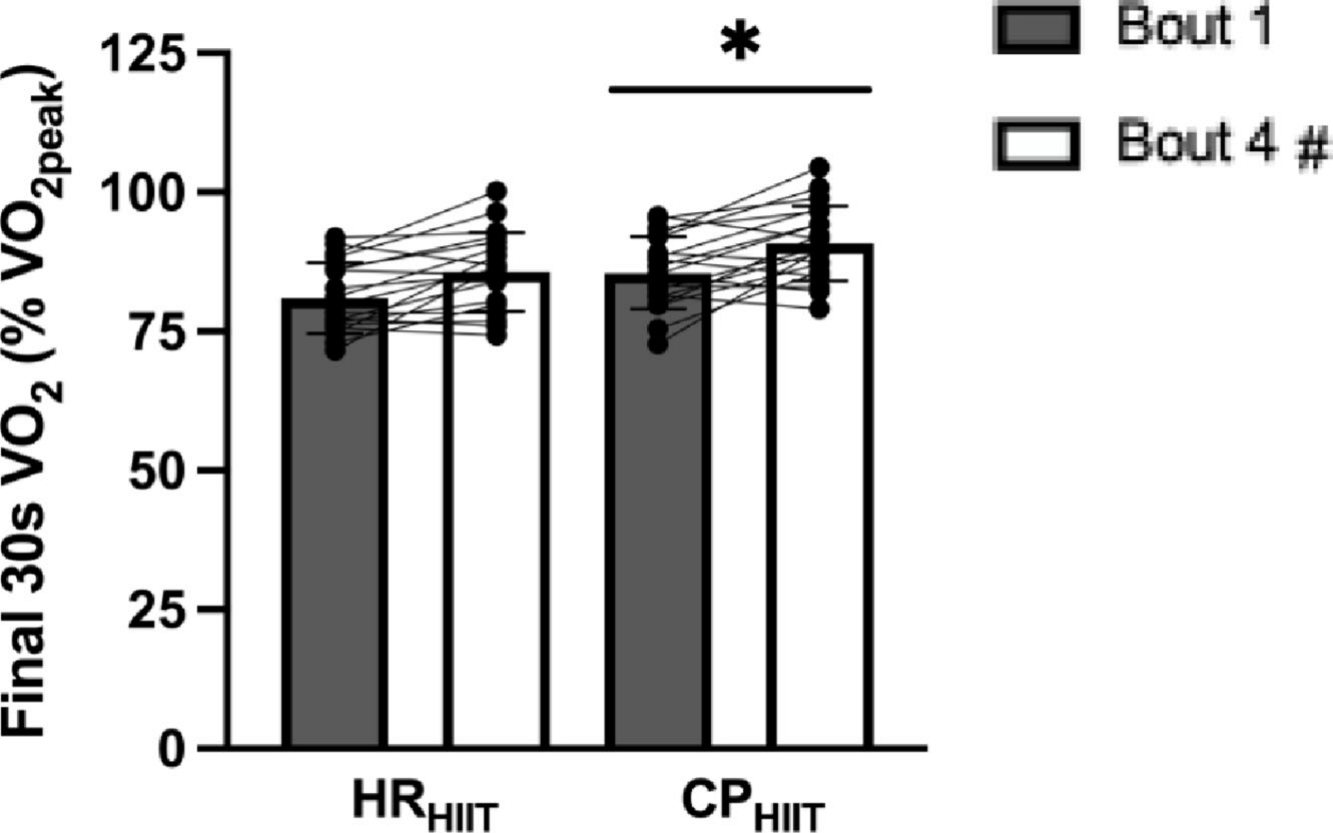
Last 30 s oxygen uptake data. Final 30 s $\dot{V}$O_2_ during Bout 1 and Bout 4 in each trial. All data are mean ± SD (*n* = 19). HR_HIIT_ = heart rate-based experimental condition; CP_HIIT_ = critical power-based experimental condition. ^*^p < 0.05 vs. HR_HIIT_, main effect for condition. ^#^*p* < 0.05 vs. Bout 1, main effect for time.

$\dot{V}$˙O_2_ over the final 30 s of each bout was higher during CP_HIIT_ vs. HR_HIIT_ when expressed in both absolute units (main effect of condition, *p* = 0.0019; η_p_^2^ = 0.95) and relative to $\dot{V}$O_2peak_ (main effect of condition, *p* = 0.0012; η_p_^2^ = 0.97, Fig. 4). $\dot{V}$O_2_ over the entire 4-minutes of each bout was higher during CP_HIIT_ vs. HR_HIIT_ (main effect of condition, *p* = 0.0007; η_p_^2^ = 0.95). There was also an interaction effect with time (*p* = 0.0011, η_p_^2^ = 0.46) such that 4-minute $\dot{V}$O_2_ was higher in CP_HIIT_ vs. HR_HIIT_ in both bouts 1 (*p* < 0.0001, *d*_*z*_ = 0.76) and 4 (*p* < 0.0001, *d*_*z*_ = 1.05). 4-minute $\dot{V}$O_2_ when expressed as a percentage of $\dot{V}$O_2peak_ was also different between conditions (*p* = 0.0005, η_p_^2^ = 0.96) and showed an interaction effect (*p* = 0.0005, η_p_^2^ = 0.49), being higher in CP_HIIT_ as compared to HR_HIIT_ in both bouts 1 (*p* = 0.019, *d*_*z*_ = 0.81) and 4 (*p* < 0.0001, *d*_*z*_ = 1.10). The $\dot{V}$˙_E_ response was similar to $\dot{V}$O_2_, such that it was higher during CP_HIIT_ vs. HR_HIIT_ (main effect of condition, *p* = 0.0007, η_p_^2^ = 0.87). There was an interaction with time (*p* = 0.0029, η_p_^2^ = 0.40), with $\dot{V}$_E_ being higher in CP_HIIT_ vs. HR_HIIT_ for bout 1 (*p* < 0.0001, *d*_*z*_ = 0.75) and bout 4 (*p* < 0.0001, *d*_*z*_ = 1.00). RER was found to be not different between conditions (*p*=0.26, η_p_^2^ = 0.20). There was also a main effect of time for final 30 s $\dot{V}$O_2_, mean $\dot{V}$O_2_, $\dot{V}$_E_, and RER across the first and fourth exercise bouts (*p* < 0.0001 for all). Respiratory gas data are summarized in Table 2. There were no differences in variability between CP_HIIT_ and HR_HIIT_ in all respiratory measures (all *p* > 0.05; see Supplementary Table S1 online).

**Table 2:** Respiratory gas data.

	CP_HIIT_		HR_HIIT_	
	Bout 1	Bout 4	Bout 1	Bout 4
30-s V̇O_2_ (L/min)^*#^	2.77 ± 0.58	2.95 ± 0.64	2.64 ± 0.62	2.79 ± 0.70
30-s VO_2_ (% $\dot{V}$O_2peak_)^*#^	86 ± 7	91 ± 7	81 ± 6	85 ± 7
4-min $\dot{V}$O_2_ (L/min)^*#^	2.35 ± 0.47^a^	2.54 ± 0.54^b^	2.26 ± 0.52	2.40 ± 0.59
4-min $\dot{V}$O_2_ (% $\dot{V}$O_2peak_)^*#^	73 ± 7^a^	78 ± 6^b^	70 ± 6	74 ± 5
$\dot{V}$_E_ (L/min)^*#^	62.5 ± 11.3^a^	72.2 ± 16.2^b^	58.9 ± 12.8	65.3 ± 17.2
RER^#^	0.95 ± 0.04	0.86 ± 0.03	0.95 ± 0.05	0.85 ± 0.03

All data are mean ± SD (*n* = 19). 30 s $\dot{V}$O_2_ = exercise oxygen uptake in the final 30 s of exercise; 4 min $\dot{V}$O_2_ = mean exercise oxygen uptake from 0 to 4 min of exercise; $\dot{V}$O_2peak_ = peak exercise oxygen consumption; $\dot{V}$_E_ = mean ventilation from 0 to 4 min of exercise; RER = respiratory exchange ratio from 0 to 4 min of exercise; HR_HIIT_ = heart rate-based experimental condition; CP_HIIT_ = critical power-based experimental condition.

^*^p < 0.05 main effect for condition.

^#^*p* < 0.05 vs. Bout 1, main effect for time.

^a^*p*<0.05 vs. Bout 1 HR_HIIT_.

^b^*p*<0.05 vs. Bout 4 HR_HIIT_.

**Fig. 5:**
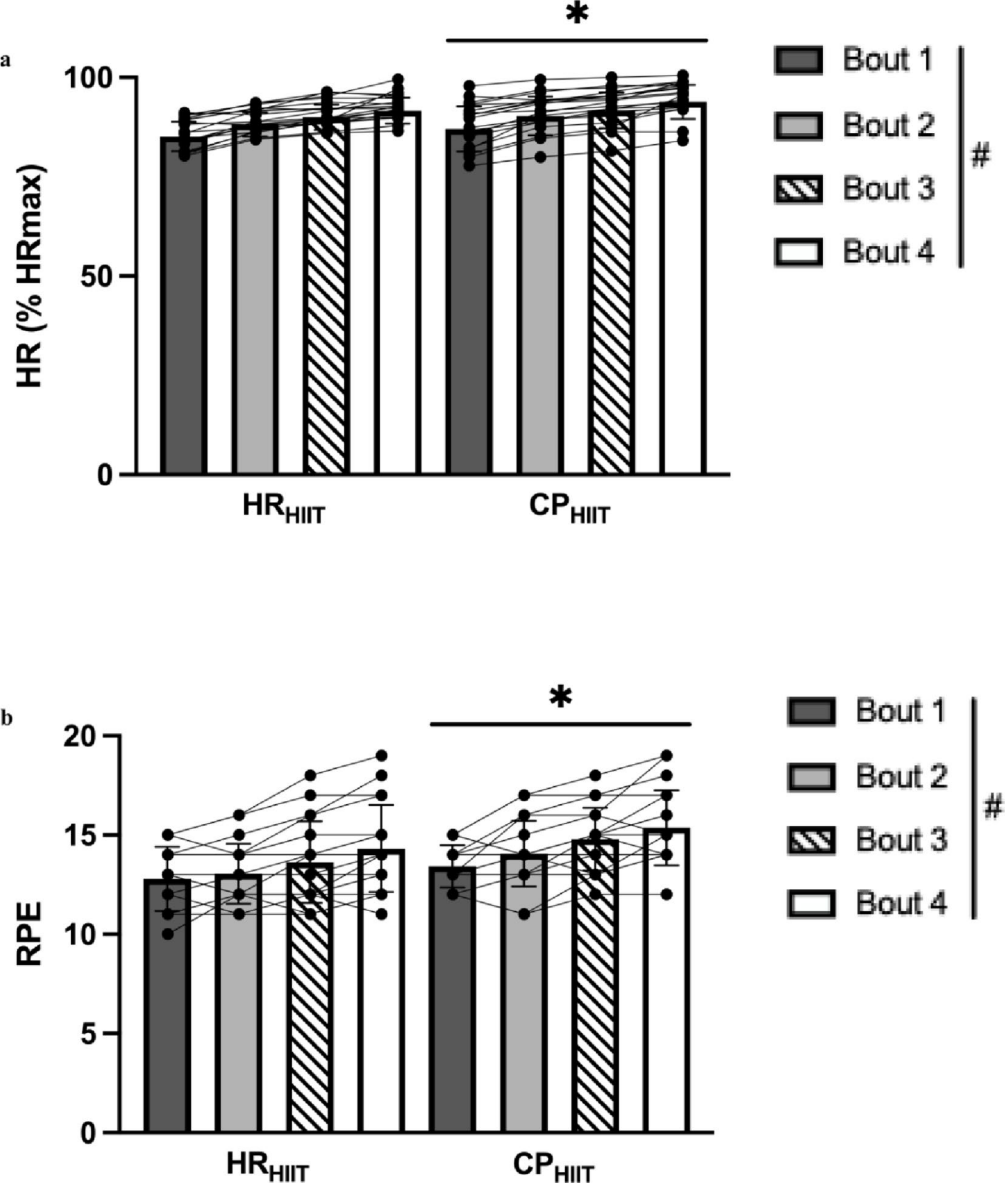
Heart rate and rating of perceived exertion data. (**a**) Peak exercise heart rate relative to individual maximal exercise heart rate between experimental conditions. (**b**) Rating of perceived exertion at the end of each bout during experimental HIIT trials. Data are reported as mean ± SD (*n* = 18 for CP_HIIT_ in Bout 2 only, *n* = 19 for rest). HR = heart rate; HR_max_ = maximal exercise heart rate; RPE = rating of perceived exertion; HR_HIIT_ = heart rate-based experimental condition; CP_HIIT_ = critical power-based experimental condition. ^*^p < 0.05 vs. HR_HIIT,_ main effect for condition. ^#^*p* < 0.05 main effect for time.

There was a main effect of condition for mean exercise HR across all bouts (HR_mean_), being higher in CP_HIIT_ vs. HR_HIIT_ when expressed in both absolute units (*p* = 0.030) and as a percentage of HR_max_ (*p* = 0.031). There was a main effect of condition for HR_peak_ when expressed in absolute units (*p* = 0.022) as well as a percentage of HR_max_ (*p* = 0.024), both of which were higher in CP_HIIT_ vs. HR_HIIT_. Notably, all participants reached a HR_peak_ of 80–90% individual HR_max_ by the end of the first bout in HR_HIIT_ (Fig. 5A). There was a main effect of time for HR_mean_, %max HR_mean_, HR_peak_, and %max HR_peak_ across all exercise bouts (*p* < 0.0001 for all). All HR data are summarized in Table 3. Variability in HRmean was greater in CP_HIIT_ vs. HR_HIIT_ in bouts 3 (10 vs. 7 beats/min, *p* = 0.021) and 4 (10 vs. 7 beats/min, *p* = 0.031) when expressed in absolute units, and in bout 2 when relative to individual HR_max_ (5 vs. 3% HR_max_, *p* = 0.044). Variability was not different for the remaining bouts for HR_mean_ and %max HR_mean_, nor in any bout for either HR_peak_ or %max HR_peak_ (all *p* > 0.05; See Supplementary Table S2 online).

**Table 3:** Exercise heart rate data.

		Bout 1	Bout 2	Bout 3	Bout 4
HR_mean_ (beats/min)^*#^	CP_HIIT_	149 ± 14	155 ± 12	159 ± 11	162 ± 11
	HR_HIIT_	146 ± 11	152 ± 9	155 ± 8	159 ± 8
HR_mean_ (% HR_max_)^*#^	CP_HIIT_	80 ± 7	83 ± 5	85 ± 5	87 ± 5
	HR_HIIT_	78 ± 5	82 ± 4	83 ± 3	85 ± 3
HR_peak_ (beats/min)^*#^	CP_HIIT_	162 ± 12	168 ± 11	171 ± 11	175 ± 10
	HR_HIIT_	159 ± 9	165 ± 8	168 ± 8	171 ± 8
HR_peak_ (% HR_max_)^*#^	CP_HIIT_	87 ± 6	90 ± 5	92 ± 5	94 ± 4
	HR_HIIT_	85 ± 4	88 ± 3	90 ± 3	92 ± 3

All data are mean ± SD (*n* = 18 for CP_HIIT_ in Bout 2 only, *n* = 19 for rest). HR_mean_ = mean exercise heart rate across 0–4 min of exercise; HR_peak_ = peak exercise heart rate; HR_max_ = maximal exercise heart rate; HR_HIIT_ = heart rate-based experimental condition; CP_HIIT_ = critical power-based experimental condition.

^*^p < 0.05 vs. HR_HIIT_, main effect for condition.

^#^*p* < 0.05 main effect for time.

### RPE

RPE was higher in CP_HIIT_ compared to HR_HIIT_ (14 ± 2 vs. 13 ± 2, main effect of condition, *p* = 0.0034; η_p_^2^ = 0.63, Fig. 5B), and increased over the course of the four exercise bouts (main effect of time, *p* < 0.0001). There was no interaction effect (*p* = 0.28), and no differences in variability for any bout (all *p* > 0.05; Table S2).

## Discussion

The primary novel finding of this study was that — in contrast to our hypothesis — there was no difference in the variability in [ΔBLa^−^] between a CP-based HIIT trial compared to one anchored to % HR_max_. Participants, however, maintained a higher workload during CP_HIIT_ as compared to HR_HIIT_, and this was associated with higher exercise $\dot{V}$O_2_, $\dot{V}$_E_, HR, and RPE. Given the higher power output and acute physiological stress, it is possible that HIIT anchored to CP rather than HRmax could translate into different training-induced responses.

### Variability of [ΔBLa^−^]

Only one previous study has examined the acute metabolic responses to a HIIT protocol anchored to CP as compared to a traditional physiological anchor^17^. The authors found reduced variability in both mean and peak exercise [BLa^−^] in 10 healthy, recreationally active participants who performed a 5 × 3-minute HIIT trial with workload intensity set at 110% CP, as compared to 85% $\dot{V}$O_2max_^17^. In contrast, we found no difference in the variability of [ΔBLa^−^] in a similar group of 19 individuals who performed a 4 × 4-min HIIT session at a workload corresponding to CP + 10% W′ or >80% HR_max_. These divergent findings could be related to the difference in prescribed workloads for the CP-based and traditional physiological anchor-between the two studies and the subsequent effects on [BLa^−^] responses.

Changes in [BLa^−^] during exercise are intensity dependent. Relatively intense but submaximal, constant-load exercise at workloads around CP elicit BLa^−^ concentrations of ~ 5–8 mM^8,27,28^. In contrast, intense exercise above CP typically results in [BLa^−^] ranging from 10 to 20 + mM depending on the specific protocol, i.e. ramp-incremental tests to maximum volitional effort vs. ‘all out’ sprints^29^. The peak [BLa^−^] responses to CP_HIIT_ and HR_HIIT_ of ~ 7–8 mM in the present study aligns with the expected range in response to exercise performed around CP^8,27,28^. The mean absolute workloads (192 ± 39 vs. 180 ± 43 W, respectively) and their proximity to CP (103 vs. 97%, respectively) were similar between the two trials. The peak oxygen uptake responses to these workloads (in the final 30 s of Bout 4) were 91 ± 7 and 85 ± 7% of $\dot{V}$O_2peak_ for CP_HIIT_ and HR_HIIT_, respectively (Table 2). The relative intensity and absolute workload of the CP_HIIT_ and HR_HIIT_ trials may thus have been too similar for any differences in the variability of [ΔBLa^−^] to be observed. In contrast, the difference in the workload of the traditional compared to the CP condition in the study by Meyler et al.^17^ was higher than in the present study. The intended intensity in the traditional trial was ~ 85% of VO_2max_ and the achieved value of 100 ± 5% VO_2max_^17^. As a result, the workload of the traditional trial in that study^17^ was considerably higher than the CP-based trial (228 ± 23 vs. 190 ± 30 W), which resulted in higher exercise [BLa^−^] (~ 11 vs. 7 mM, respectively) and this may have contributed to the difference in [BLa^−^] variability observed.

The higher [BLa^−^] response to the traditional relative to the CP trial in the study by Meyler et al.^17^ could have influenced the outcome of the [BLa^−^] variability through heteroscedasticity, which refers to when the variability of a dataset (measured in standard deviations [SD]) changes with the size of the mean, such that groups with larger means have proportionally larger SDs. This is relevant as authors assessed variability in [BLa^−^] by comparing the SDs of the means in each condition using the F-ratio^17^. It is possible that the higher SD of the traditional group that was the basis of significant finding reported may not have entirely been due to higher metabolic variability relative to the CP-based condition per se, but rather due to the SD potentially ‘scaling’ alongside the higher mean. The Levene test, which was used to assess variability in the present study, is robust to heteroscedasticity^30^. Also, in the study by Meyler et al.^17^, only 20% of the participants completed the traditional (% $\dot{V}$O_2max_) trial, resulting in a lower number of observations as compared to the CP-based trial, which all participants completed (as in the present study). While the authors did perform a sensitivity analysis to avoid missing data biasing conclusions, no test for normality was reported^17^. In the present study, all data were tested for normality prior to analysis via a Shapiro-Wilks test. While the data followed normal distributions, the Levene test used to compare variability in [ΔBLa^−^] is also robust to non-normality should it have occurred^31^.

### Verification of HR-based exercise prescription

The prescription of exercise intensity was an integral component of this study design, particularly with respect HR_HIIT_, and the choice of an exercise intensity eliciting a peak HR response of ≥ 80% HR_max_ was made after careful consideration. A minimum intensity of 80% HR_max_ falls just above the lower bound of the range of HR responses at which exercise intensity is classically considered to be “vigorous”, or appropriate for HIIT per traditional guidelines^3^. Therefore, it not only serves as an appropriate intensity for HIIT, but was thought (and ultimately demonstrated to be) appropriate to ensure that all participants were able to complete a 4 × 4-min trial using this metric. As noted above, this was not the case in the study by Meyler et al.^17^, in which only 20% of participants completed the traditional trial. Also as previously noted, the peak exercise $\dot{V}$O_2_ response during the traditional condition exceeded the prescribed intensity of 85% $\dot{V}$O_2max_, and elicited a mean exercise VO_2_ of 97 ± 5% $\dot{V}$O_2max_. Therefore, in the present study, ensuring that the HR response elicited from the HR_HIIT_ workload elicited the desired response of 80–90% HR_max_ by the end of the first exercise bout was paramount. This was achieved through assessing the HR responses to exercise at 60% PPO during the HR_HIIT_ familiarization trial, which if falling outside the range of 80–90% HR_max_ was adjusted to elicit the desired response in the subsequent experimental trial.

Employing a familiarization trial to verify the intended exercise intensity was done as an alternative to the common methods of prescribing exercise intensity relative to HR_max_. When prescribing exercise at intensities relative to HR_max_ (and $\dot{V}$O_2max_), it is often assumed that the desired response for a given constant intensity is predictable from a ramp-incremental test^32^. This is based on the assumption that both HR and $\dot{V}$O_2_ increase linearly with exercise intensity until maximum, but this is not the case at intensities above the lactate threshold (LT)^32–34^. Constant work rate exercise at intensities above the LT (such as bouts in typical HIIT protocols) prescribed in this manner would result in higher-than-expected exercise responses^32^. This effect could have influenced the $\dot{V}$O_2_ response to the traditional condition in the study by Meyler and colleagues, as the authors explained that the workloads were extrapolated from the $\dot{V}$O_2_-intensity relationship in their ramp-incremental test^17^. Researchers should consider the use of verification phases when using these traditionally based models to ensure the desired response to exercise is elicited.

### Feasibility of CP-based exercise

The individual workloads prescribed for HR_HIIT_ in the present study, which were intended to elicit HR responses of ≥ 80% HR_max_, varied widely around individual CP values, corresponding to 77–111% of CP. This is a demonstration of “domain overlap”, a common phenomenon which reflects the varied exercise responses to exercise prescribed using maximal physiological measures as exercise intensity anchors^12–14^. CP_HIIT_ in contrast did not demonstrate any overlap, given the nature of metabolic threshold and the fact that it was anchored just above CP (101–106% of CP). There is some work to suggest that carefully considering workload proximity to CP when prescribing exercise intensity may help optimize exercise training outcomes compared to traditional methods of prescription. Collins^35^ examined healthy but inactive adults who underwent 8 weeks of either moderate-intensity continuous training or HIIT at fixed percentages of $\dot{V}$O_2max_ PPO three times per week. The authors found that training-induced changes in CP were more strongly correlated to the intensity of the exercise training program relative to individual CP vs. % $\dot{V}$O_2max_^35^. This suggests that anchoring exercise intensity directly to CP may result in greater training induced adaptations.

In the present study, participants experienced greater cardiorespiratory (Tables 2 and 3) and perceived stress in CP_HIIT_ compared to HR_HIIT_ on account of a higher workload (Fig. 2A). It is well established that exercising at higher intensities results in greater skeletal muscle and cardiovascular adaptations^36^. Exercise at higher intensities has been shown to increase both mRNA expression and protein synthesis of peroxisome proliferator-activated receptor γ 1‐α (PGC-1α), the “master regulator” of mitochondrial biogenesis^37,38^. Furthermore, greater increase in $\dot{V}$O_2max_ in response to interventions at higher exercise intensities have been reported even when matched for work with lower intensity protocols^39,40^. The higher workload in CP_HIIT_ was reflected through higher cardiorespiratory stress and perceived exertion compared to HR_HIIT_, and could translate to greater training-induced adaptations over a long-term improvements. Such findings would not be without precedent, as other threshold-based training interventions have been shown to elicit greater adaptations compared to traditional ones^15^, although it should be noted that others have reported no differences^16^. While expanding on such a topic is outside of the scope of the present study due to the acute nature of the work, future research should examine whether long-term CP-based HIIT interventions elicit greater physiological responses compared to traditional approaches.

### Strengths and limitations

A strength of the present study was the sample size, which was almost double that of the single comparable study available in Meyler et al.^17^, and based on an a priori estimate to detect a difference in [ΔBLa^−^] variability with a large effect size. The true effect size, however, may have been smaller than hypothesized and necessitate a larger sample. Another strength was that all of our participants fully completed both the CP_HIIT_ and HR_HIIT_ trials, which is relevant as the previous study by Meyler and colleagues^17^ saw only a 20% completion rate in the traditional ($\dot{V}$O_2_-based) trial. This enabled a more robust comparison of the CP_HIIT_ and HR_HIIT_ approaches, as all participants competed both trials. These data also demonstrate that the CP + 10% approach was feasible for workload prescription when applied to a 4 × 4-min HIIT protocol, showcasing the utility of a CP-based approach as compared maximum traditional physiological anchors. The familiarization trials also ensured that the desired exercise HR responses were elicited during the HR_HIIT_ trials. The study protocol was also registered prior to data collection.

A limitation of this study was in prescribing CP_HIIT_ at a relatively conservative intensity of CP + 10% W′, given research has shown that a substantial amount of W′ can be reconstituted after similar periods of recovery^41,42^. This choice was made in an effort to ensure that all participants were able to complete the exercise trials, however it is probable that all bouts could have been performed to completion at substantially higher percentages of W′ exhaustion, which future studies should explore. Another limitation was in not ensuring a W′ SE of < 10% in all participants, potentially influencing the calculated intensities for CP_HIIT_ which were prescribed as CP + 10% W′. This is also likely the reason behind the linear work-time model providing the best fit for all participants. We also acknowledge that there may have been increased variability in physiological responses on account of the inclusion of participants from both Tiers 1 and 2 of the McKay classification framework. This may have also contributed to potential learning and training effects during testing. Handheld point of care capillary BLa^−^ sampling devices were used in this study to both mimic the work of Meyler et al.^17^ and given the practicality of this tool in the field. However, reports of the accuracy and validity of such devices are mixed^43,44^. More invasive studies that incorporate skeletal muscle biopsy sampling and measure relevant metabolites (e.g. muscle lactate and phosphocreatine) may offer a clearer lens into the variability of metabolic responses between traditional and threshold-based exercise.

## Conclusion

Anchoring exercise intensity to CP did not reduce variability of [ΔBLa^−^] compared to a traditional, HR-based approach during traditional HIIT. However, the CP-based method did result in participants achieving a higher workload and therefore resulting in higher cardiorespiratory and perceived stress compared to the HR-based protocol. These results add to the discourse surrounding the influence of metabolic threshold-based training on the variability in exercise responses. Furthermore, this study also provides evidence supporting that such training interventions may result in divergent training-induced responses compared to traditional exercise protocols.

## Supplementary Information

Below is the link to the electronic supplementary material.

Supplementary Material 1

## Data Availability

Deidentified participant data that underlie the results presented in this article will be available to researchers upon review and approval of reasonable requests starting six months after manuscript publication. Proposals should be sent to the corresponding author.
