# Supplementary material for: Cardiorespiratory and metabolic stress responses to acute high-intensity interval training anchored to critical power or maximal heart rate
Source: Sci Rep. 2025 Dec 29;15:44700. doi: 10.1038/s41598-025-28231-y (PMC12749765; doi:10.1038/s41598-025-28231-y)
Supplement: Supplementary file 1 — Supplementary Material 1 [file 41598_2025_28231_MOESM1_ESM.pdf]

Cardiorespiratory and metabolic stress responses to acute high-intensity interval training anchored to critical power or maximal heart rate

Jack Bone[1], Douglas L. Richards[2], Martin J. Gibala[1]*

[1]Department of Kinesiology, McMaster University, Hamilton, Ontario, Canada.

[2]Department of Medicine, McMaster University, Hamilton, Ontario, Canada.

Contact Information

Jack Bone, M.Sc.

bonej@mcmaster.ca

Douglas L. Richards, M.D.

Richad10@mcmaster.ca

*Corresponding author: Martin J. Gibala, Ph.D.

Email: gibalam@mcmaster.ca

Supplemental Table S1: **Variability in respiratory gas data**

	**CP_{HIIT}**		**HR_{HIIT}**	
	Bout 1	Bout 4	Bout 1	Bout 4
**30-s $\dot{V}O_2$ (L/min)**	0.47±0.32	0.53±0.33	0.51±0.34	0.58±0.35
**30-s VO_2 (% $\dot{V}O_{2peak}$)**	6±4	5±4	5±3	5±4
**4-min $\dot{V}O_2$ (L/min)**	0.41 (0.09-0.52)	0.44±0.31	0.33 (0.24-0.54)	0.49±0.32
**4-min $\dot{V}O_2$ (% $\dot{V}O_{2peak}$)**	6±3	5±3	5±3	4±3
**$\dot{V}_E$ (L/min)**	8.4 (6.3-11.1)	12.9±9.4	8.5 (2.7-13.8)	13.5±10.2
**RER**	0.02 (0.01-0.03)	0.02±0.02	0.02 (0.01-0.03)	0.02±0.02

All Bout 4 as well as Bout 1 for 30-s $\dot{V}O_2$ (L/min), 30-s $\dot{V}O_2$ (% $\dot{V}O_{2peak}$), and 4-min $\dot{V}O_2$ (% $\dot{V}O_{2peak}$) are mean ± SD (n = 19). Bout 1 data for 30-s $\dot{V}O_2$ (L/min), 30-s $\dot{V}O_2$ (% $\dot{V}O_{2peak}$) and 4-min $\dot{V}O_2$ (% $\dot{V}O_{2peak}$) are median (interquartile range). 30-s $\dot{V}O_2$ = exercise oxygen uptake in the final 30 seconds of exercise; 4 min $\dot{V}O_2$ = mean exercise oxygen uptake from 0-4 minutes of exercise; $\dot{V}O_{2peak}$ = peak exercise oxygen consumption; $\dot{V}_E$ = mean ventilation from 0-4 minutes of exercise; RER = respiratory exchange ratio from 0-4 minute of exercise; HR_{HIIT} = heart rate-based experimental condition; CP_{HIIT} = critical power-based experimental condition.

Supplemental Table S2: **Variability in heart rate and perceived exertion data**

		**Bout 1**	**Bout 2**	**Bout 3**	**Bout 4**
**HR_{mean}**	**CP_{HIIT}**	12±7	10±6	10 (6-15)[c]	6±3[c]
**(beats/min)**	**HR_{HIIT}**	8±6	7±5	8 (3-11)	4±2
**HR_{mean}**	**CP_{HIIT}**	6±3	5±3[c]	4±3	3 (2-5)
**(% HR_{max})**	**HR_{HIIT}**	4±2	3±2	3±2	1 (1-3)
**HR_{peak}**	**CP_{HIIT}**	10±6	9±5	10±4	9±5
**(beats/min)**	**HR_{HIIT}**	7±5	7±4	7±4	7±4
**HR_{peak}**	**CP_{HIIT}**	5±3	4±3	3 (1-5)	3 (2-4)
**(% HR_{max})**	**HR_{HIIT}**	3±2	3±1	2 (1-4)	3 (1-3)
**RPE**	**CP_{HIIT}**	0.6 (0.5-1.4)	1.1 (0.1-1.9)	1.2 (0.2-1.8)	1.4 (0.6-1.6)
	**HR_{HIIT}**	1.2 (0.5-2.0)	1.1 (0.9-1.5)	1.6 (1-2.4)	1.3 (0.7-2.5)

All HR-related data in Bouts 1 and 2 are mean±SD, along with HR_{peak} (beats/min) for Bouts 3 and 4, HR_{mean} (% HR_{max}) for Bout 3, and HR_{mean} (beats/min) for Bout 4 (n = 18 for CP_{HIIT} in Bout 2 only, n = 19 for rest). All RPE data as well as HR_{peak} (% HR_{maz}) for Bouts 3 and 4, HR_{mean} (beats/min) for Bout 3, and HR_{mean} (% HR_{max}) for Bou4 4 are median (interquartile range). HR_{mean} = mean exercise heart rate across 0-4 minute of exercise; HR_{peak} = peak exercise heart rate; HR_{max} = maximal exercise heart rate; RPE = rate of perceived exertion; HR_{HIIT} = heart rate-based experimental condition; CP_{HIIT} = critical power-based experimental condition.
[c]$p<0.05$ vs same Bout HR_{HIIT}.
